# Clinical efficacy and tendon integrity of patients with subscapularis tear by the technique of arthroscopic single external row repair

**DOI:** 10.3389/fmed.2023.1167158

**Published:** 2023-07-25

**Authors:** Weipeng Zheng, Rui Weng, Xiaohang Wu, Zhijun Liu, Zhihao Liao, Sheng Chen, Suming Zheng, Zhiyong Yi, Xudong Huang, Hewei Wei

**Affiliations:** ^1^The Third Affiliated Hospital of Guangzhou University of Traditional Chinese Medicine, Guangzhou, China; ^2^Guangdong Research Institute for Orthopedics and Traumatology of Chinese Medicine, Guangzhou, China; ^3^Guangzhou University of Traditional Chinese Medicine, Guangzhou, China

**Keywords:** arthroscopic, subscapularis tear, single-row repair technique, SwiveLock^®^ C external row anchor fixation, efficacy

## Abstract

**Background:**

With the development of arthroscopic technology and equipment, arthroscopy can effectively repair the tear of the subscapular muscle. However, it is difficult to expose the subscapular muscle and operate it under a microscope. In this study, the SwiveLock^®^ C external row anchor under arthroscopy was applied to repair the tear of the subscapular muscle in a single row, which is relatively easy to operate with reliable suture and fixation, and its efficacy was evaluated.

**Purpose:**

This study aimed to assess the clinical efficacy and the tendon integrity of patients who had subscapularis tears by adopting the single-row repair technique with a SwiveLock^®^ C external row anchor.

**Methods:**

Patients who had the subscapular muscle tear either with or without retraction were included, and their follow-up time was at least 1 year. The degree of tendon injury was examined by magnetic resonance imaging (MRI) and confirmed by arthroscopy. The tendon was repaired in an arthroscopic manner by utilizing the single-row technique at the medial margin of the lesser tuberosity. One double-loaded suture SwiveLock^®^ C anchor was applied to achieve a strong fixation between the footprint and tendon. The range of motion, pain visual simulation score, American Shoulder and Elbow Surgeons (ASES) score, and Constant score of shoulder joint were evaluated for each patient before the operation, 3 months after the operation, and at least 1 year after the operation.

**Results:**

In total, 110 patients, including 31 males and 79 females, with an average age of 68.28 ± 8.73 years were included. Arthroscopic repair of the subscapular tendon with SwiveLock^®^ C external anchor can effectively improve the range of motion of the shoulder joint. At the last follow-up, the forward flexion of the shoulder joint increased from 88.97 ± 26.33° to 138.38 ± 26.48° (*P* < 0.05), the abduction range increased from 88.86 ± 25.27° to 137.78 ± 25.64° (*P* < 0.05), the external rotation range increased from 46.37 ± 14.48° to 66.49 ± 14.15° (*P* < 0.05), and the internal rotation range increased from 40.03 ± 9.01° to 57.55 ± 7.43° (*P* < 0.05). The clinical effect is obvious. The constant shoulder joint score increased from 40.14 ± 15.07 to 81.75 ± 11.00 (*P* < 0.05), the ASES score increased from 37.88 ± 13.24 to 82.01 ± 9.65 (*P* < 0.05), and the visual analog scale score decreased from 5.05 ± 2.11 to 1.01 ± 0.85 (*P* < 0.05). In the 6th month after the operation, two cases (1.81%) were confirmed to have re-tears via MRI.

**Conclusion:**

In this study, we repaired the subscapularis muscle with a single-row technique fixed by SwiveLock^®^ C anchor and FiberWire^®^ sutures and evaluated its efficacy. The results showed that the clinical effect of single-row arthroscopic repair was satisfactory and that reliable tendon healing could be achieved.

## Introduction

Previously, large rotator cuff tears were repaired by open procedures. However, at present, surgeons have the option to use arthroscopic rotator cuff repair techniques (1, 2). Arthroscopic supraspinatus and infraspinatus tendon repair techniques are well-established, but the arthroscopic repair technique for the subscapularis tendon is a recent development. Consequently, subscapularis repair under arthroscopy has received increased attention. Currently, the incidence of subscapularis tears is 31.4% among all arthroscopic rotator cuff repairs (3). However, arthroscopic repair of the subscapularis tendon is quite a difficult operation among all rotator cuff surgeries, particularly in the case of a chronic subscapularis tear with retraction. Owing to the special anatomical structure and biomechanical characteristics of the subscapularis muscle, in arthroscopic repair of the subscapularis muscle, there are greater difficulties and challenges in terms of exposure and microscopic operation compared with supraspinatus and infraspinatus muscle tears. There are several reasons for the difficulty of the arthroscopic repair of the subscapularis tendon. First, if the subscapular tendon retracts to the glenoid, it will increase the difficulty of conventional arthroscopic exploration and tendon reduction. Second, the coracoid process and conjoined tendons adjacent to the neurovascular structure limit joint activity. Finally, because of the close subscapularis space covering the subscapularis tendon, surgeons find it difficult to operate arthroscopic instruments (4, 5).

With the development of arthroscopic techniques and equipment, arthroscopic repair of subscapularis tears, due to its inherent superiority, is assumed to be possibly better than open repair because it is a minimally invasive surgery and can treat concomitant posterior–superior rotator cuff tears at the same time. At present, the repair methods mainly include single-row sutures and double-row sutures. Due to the narrow space in front of the shoulder joint and the unclear visual field, the technical operation of double-row anchors is more difficult, the operation time is long, and multiple anchors and thread knots are easy to cause postoperative adhesion or new impact. The disadvantage of the general single-row anchor nail fixation technique is that the rotator cuff is in point contact with the bone surface, which cannot achieve anatomical healing, and its stress is relatively concentrated, which is easy to cause re-tearing. In this study, a single-row repair and suture technique was used to repair the tear of the subscapular muscle under an arthroscope with an external anchor. The essence is to use two FiberWire^®^ sutures (Arthrex, Florida, America) to form two locking loops at the broken end of the tendon and a single SwiveLock^®^ C anchor (Arthrex, Florida, America) to fix it in the cortical bone area outside the small nodule healing area to achieve surface-to-surface contact between the subscapular tendon and the “footprint.”

The FiberWire^®^ suture is a new generation of non-absorbable mixed high-polymer woven sutures approved by the FDA in the United States. The suture has excellent tensile strength, flexibility, plasticity, and excellent tissue compatibility, which is conducive to tissue healing. Surgeons can exert greater force to tighten and fix the suture without worrying about additional risks such as prolonged surgery time and iatrogenic injury caused by breakage. In addition, the high strength of the suture can withstand greater loads brought about by postoperative rehabilitation exercises, thus avoiding fixation failure and tissue nonunion caused by suture breakage due to low suture strength under the same load conditions (6). The SwiveLock^®^ C is a fully threaded, knotless anchor designed for use with FiberWire^®^ sutures in rotator cuff repair by applying the SpeedFix™, SutureBridge™, or SpeedBridge™ techniques. The Labral SwiveLock was available for rotator cuff repair. It provides maximum pull-out and insertion strength while saving time. The simple knotless technique consists of passing FiberWire^®^ suture through tissue, loading them through the closed SwiveLock eyelet, which is then inserted into a bone socket. Tension is visualized, adjusted, and locked into position with the SwiveLock anchor body. SwiveLocks are cannulated and vented to minimize material and allow for potential bony ingrowth (7, 8). Therefore, we applied FiberWire^®^ sutures and SwiveLock^®^ C to repair subscapularis tears. This study aims to evaluate the clinical efficacy and tendon integrity of a subscapularis tear by using the arthroscopic single-row repair technique.

## Methods

The study was approved by the Ethics Committee of the Third Affiliated Hospital of Guangzhou University of Traditional Chinese Medicine [Approval no.: KY (2019) 002], and the participating patients provided written informed consent.

### Patient selection

From April 2016 to October 2021, we consecutively assessed 805 patients who were confirmed with rotator cuff tears during arthroscopic rotator cuff repair at a single institution. The indication for operative treatment was symptomatic isolated or combined partial-thickness or full-thickness subscapularis tears. This study targeted patients who were suffering from full- or partial-thickness subscapularis tears with partial articular subscapularis tendon avulsion and an inadequate level of tendon retraction, who had undergone arthroscopic single-row repair, and who had been followed up for at least 1 year after surgery.

The inclusion criteria were as follows: Patients with trauma or chronic cumulative injury who have clinical symptoms of a shoulder injury such as pain and limited activity, who received non-operative treatment that was ineffective (after 3 months of conservative treatment), and whose preoperative physical examinations conform to the performance of subscapular muscle tear, such as bear hug test (+) and belly press test (+); magnetic resonance imaging (MRI) examination showed partial- or full-thickness tear of the subscapular muscle, no tendon retraction, or retraction to the medial side of the footprint of the humeral head, but not to the joint fossa, with or without other rotator cuff tendon injuries; and those who are willing to follow-up for a period of more than 1 year and cooperate during the experiment.

The exclusion criteria were as follows: previous history of other shoulder surgery or joint infection; patients with previous upper limb nerve injury or deltoid dysfunction and severe shoulder instability; those for whom the standard for fat infiltration in the subscapular muscle is Goutellier type IV according to MRI images; irreparable tear of the subscapular muscle or severe shoulder osteoarthritis; poor cardiopulmonary function for surgery; patients with a history of mental illness and who cannot cooperate well; and patients who have no intention of rehabilitation or cannot be followed up.

### Clinical and radiological evaluations

Plain radiographs, such as axial, anteroposterior, and supraspinatus outlet views, were acquired for all patients to assess the glenohumeral osteoarthritis presence and superior migration of the humeral head. MRI was performed preoperatively to evaluate the level of tendon retraction, the related rotator cuff pathologies, and the fatty infiltration status of the subscapularis muscle.

**Figure 1:**
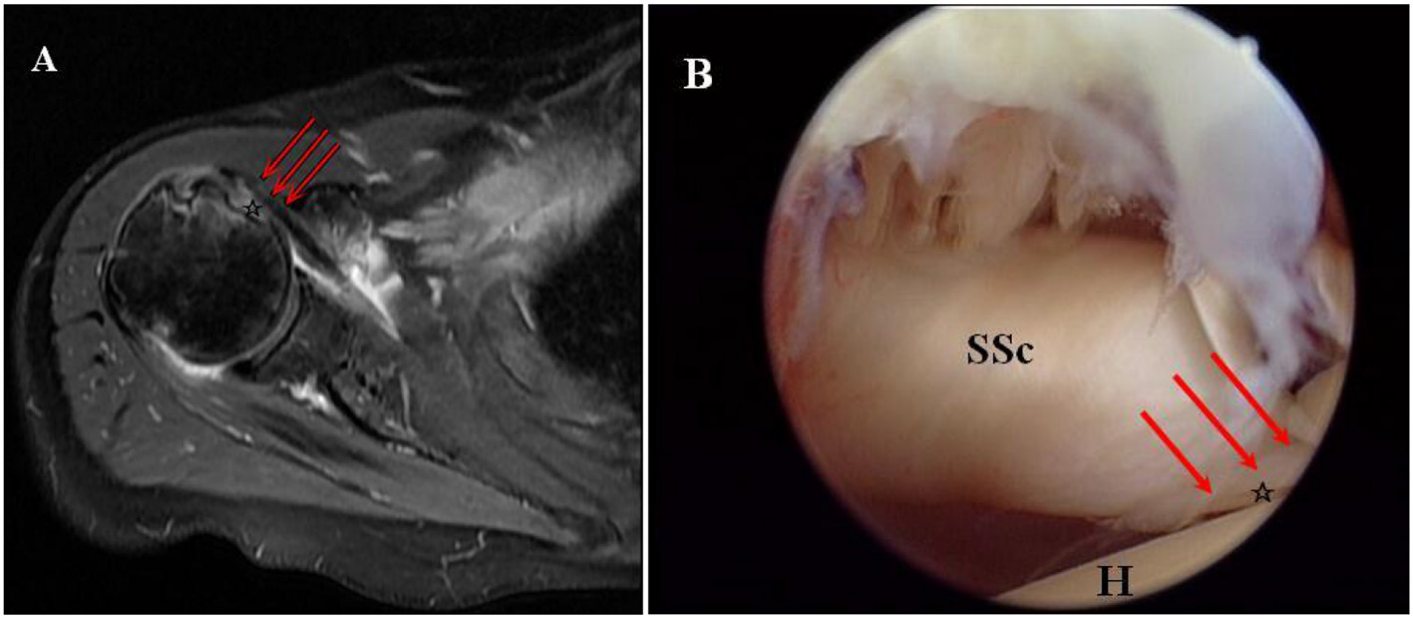
Torn subscapularis tendon without retraction in the right shoulder of a female patient aged 64 years. **(A)** Torn subscapularis tendon (asterisk) without retraction showed on preoperative axial T2-weighted MRI. **(B)** Non-retraction of the torn subscapularis tendon (asterisk) was detected according to an arthroscopic intra-articular view from the posterior portal (SSc, subscapularis; H, humeral head).

The tendon retraction level was measured on axial images according to the Patte classification and then confirmed during the operation (Figure 1A) (9). The fatty infiltration of the subscapularis muscle was divided into three grades from I to III according to the Fuchs classification, which got modified for MRI according to the Goutallier classification (10, 11). Fuchs grade I corresponds to Goutallier grades 0 and I, Fuchs grade II is equal to Goutallier grade II, and Fuchs grade III is equal to Goutallier grades III and IV. Two shoulder orthopedic surgeons who were blinded to patient's personal details performed evaluations of radiologic measurements, and interobserver reliability was obtained based on the intraclass correlation coefficient (ICC). The subscapularis injuries were listed into different classifications intraoperatively according to the Lafosse classification (Figure 1B) (12). Some patients underwent MRI after surgery to evaluate the structural integrity of the repaired tendon. The tendon integrity was categorized as intact or re-tear on axial T2-weighted MRI scans (13, 14). Tendon integrity after surgery on MRI was examined by a musculoskeletal radiologist who did not participate in this research.

### Surgical technique

**Figure 2:**
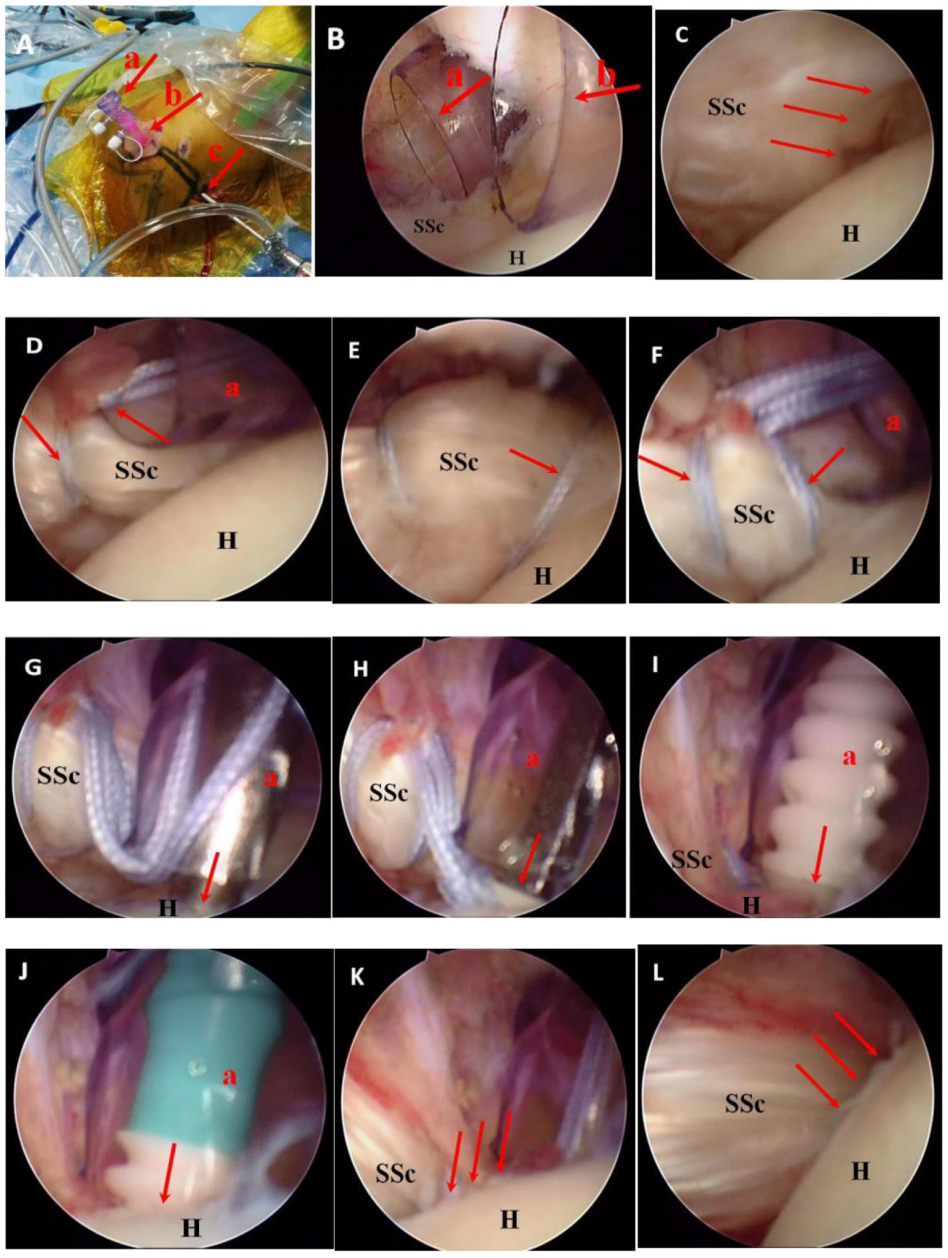
An arthroscopic view of a chronic subscapularis tear in a female patient aged 61 years. A right shoulder shown from a view of the posterolateral portal. **(A)** Approach of the glenohumeral joint: anterior portal (a), anterolateral portal (b), posterolateral portal (c). **(B)** Operation approach: anterior portal (a), anterolateral portal (b). **(C)** Complete tearing of the upper part of the subscapularis insertion. **(D)** The first FiberWire^®^ suture was passed along the upper part of the subscapularis tendon through the anterior portal. **(E)** The second FiberWire^®^ suture was passed along the external part of the subscapularis tendon in the same way. **(F)** Two FiberWire^®^ sutures were tied separately to bind the upper and external parts of the subscapularis. **(G)** An open circuit device was used to prepare a positioning bone canal above the small nodule. **(H)** The tail thread of two FiberWire^®^ sutures was pushed through the hole of a biocomposite SwiveLock^®^ C anchor (Arthrex, Florida, America) and then into the bone canal. **(I, J)** One SwiveLock^®^ C anchor was inserted at the superomedial portion of the lesser tuberosity completely, and the FiberWire^®^ is pushed into the bone bed; **(K)** The repaired footprints with low protuberances can be observed. **(L)** The subscapularis tendon after repair can be visualized above (G, glenoid; H, humeral head; SSc, subscapularis tendon).

All operations were performed by the same group of senior surgeons. The patient was placed under general anesthesia and then in the lateral decubitus position with an interscalene block. The procedure began with diagnostic arthroscopy and passed through a standard posterior viewing portal. After routine intra-articular examination, an anterior portal was made just lateral to the coracoid tip and below the coracoacromial ligament in the rotator interval. An anterolateral portal was created 2–3 cm anterior and slightly medial to the anterolateral corner of the acromion (Figures 2A, B). In the case of a chronic subscapularis tear without retraction, it is hard to identify torn tendon edges from the intra-articular view (Figure 2C). To determine the anterior edge of the subscapularis tendon with retraction, we found the so-called comma sign, or ligament structure, which consisted of the superior glenohumeral and coracohumeral ligaments (15). They were torn from the lesser tuberosity. However, they were still attached to the superolateral side of the tendon as an anatomical mark. As long as the comma sign was found, a traction suture was performed at the anterior portion of the torn tendon. Two FiberWire^®^ sutures were passed through the upper part of the subscapularis tendon and tied (Figures 2D–F) through the anterior portal and anterolateral portal. The FiberWire^®^ suture was fixed to the prepared medial margin of the lesser tuberosity with a biocomposite SwiveLock^®^ C anchor (Figures 2G–L). To ensure the subcoracoid space for arthroscopic devices, a technique of posterior lever push was adopted while the shoulder was in abduction and internal rotation (16). Due to the adoption of the anterolateral portal, the adhesion between the coracoid and subscapularis was released, and the mobility of the tendon was achieved. Such release minimized neurovascular injury, particularly on the inferior part of the tendon and the medial side of the coracoid process.

When mobility was achieved, subscapularis tendon footprints were identified and then decorticated, including the lateral part of the articular cartilage. One SwiveLock^®^ C anchor was inserted at the superomedial margin of the lesser tuberosity. The single-row technique was then applied to realize strong fixation between the footprint and the tendon. The FiberWire^®^ sutures were delivered to the external portion of the tendon fibers and tied with a suture hook from the anterior portal. Then, with the same method, the other FiberWire^®^ sutures were passed through the superior portion of the tendon and tied. One SwiveLock C^®^ anchor was normally inserted at the lateral edge of the lesser tuberosity. However, when the torn tendon was not enough to get to the lateral margin of the lesser tuberosity after mobilization, a suture anchor was inserted about 1 cm at the medial margin after decortication of the articular surface for footprint medialization. When a concomitant posterosuperior rotator cuff tear appeared, a suture bridge technique was used for repair. After decortication of the greater tuberosity, a medial row anchor was put at the junction of the articular cartilage. Sutures were delivered through the rotator cuff tendon from the anterior to posterior direction in a manner of horizontal mattress and then tied. The technique of suture bridge was utilized by putting two knotless lateral row anchors at the lateral margin of the greater tuberosity. The biceps tendon went through tenodesis or tenotomy in patients according to their age, tendon quality, labor demand, and other factors.

### Postoperative rehabilitation

After the anesthesia subsided, the practice of opening hands and clenching fists 500 times a day slowly and shoulder shrug 30–40 times a day were started. The goal of the first stage (0–4 weeks after surgery) was to reduce stress on the repaired tendon and promote early healing; therefore, it is necessary to wear a pillow-type support to fix the affected shoulder in an abduction position of 30°. The main movements were passive external rotation and forward bending swing, and external rotation activities were not allowed to exceed the neutral position. The second stage (4–8 weeks after surgery) aimed to provide certain stress stimulation to the tendons, prevent early joint adhesion, and promote the recovery of shoulder joint proprioception. Gradually, active shoulder joint exercise was started, including active external rotation and forward bending and swinging. External rotation activities were allowed to gradually exceed the neutral position. The third stage (8–12 weeks after surgery) aimed to strengthen the muscle strength of rotator cuff muscles, improve the stability of the shoulder joint and scapula, and establish a good movement mode. Initiatives were taken to carry out muscle strength training and resistance strength training of shoulder muscles. The goal of the fourth stage (12–16 weeks after surgery) was to restore daily living abilities as much as possible and return to exercise. At this stage, unrestricted activities were basically resumed (17).

### Outcome indicator

All patients completed a survey that included questions about symptom duration, the history of trauma, and other demographic information, including age, gender, and dominance of the arm. The active ROM, Visual Analogue Scale (VAS) scores of pains, American Shoulder and Elbow Surgeons (ASES), and Constant score records were assessed before surgery, 3 months after surgery, and at least 1 year after surgery. A physician assistant with 5 years of orthopedic experience who was not involved in this study evaluated the preoperative and postoperative parameters.

### Statistical analysis

The data were analyzed using SPSS 13.0 Software (SPSS, Munich, Germany), and all normal distribution data were examined by the Kolmogorov–Smirnov test. When the data were normally distributed, the independent-sample *t*-test was used for comparison between the two groups; when the data were not normally distributed, the rank sum test was used. In addition, the Wilcoxon signed rank test and *t*-test were applied to contrast continuous variables before and after surgery (18). The significance level of all tests was set at a *P*-value of < 0.05.

## Results

Among 805 patients with rotator cuff injury, 144 patients with subscapular muscle tears were selected. According to the clear exclusion criteria, we excluded 32 patients. Among them, 5 patients had a sufficient degree of full-thickness tear, atrophy, and irreparable tendon retraction; 19 patients had Lafosse I type subscapular tear; and 8 patients had severe shoulder osteoarthritis. Among the 112 patients who met the inclusion criteria, 110 were successfully followed up, and the other 2 failed to follow-up. Among them were 31 male and 79 female patients. Table 1 summarizes the demographic data before surgery. There was no pseudoparalysis before the operation. During the surgery, it was found that 78 (70.91%) patients had Lafosse classification type II subscapularis tears, and 101 (91.82%) patients had combined rotator cuff tears. In total, 5 (4.55%) patients had a full rupture of the long head of the biceps, 36 (32.73%) patients had a partial tear of the long head of the biceps, and 6 (5.45%) patients had dislocation or subluxation of the long head of the biceps. According to the preoperative Y-scan, the classification of shoulder crest is as follows: 19 (17.27%) cases in type I, 64 (58.18%) cases in type II, and 27 (24.55%) cases in type IV. Under arthroscopy, there were 36 (32.72%) cases of normal articular cartilage, 21 (19.09%) cases of type I degree injury, 29 (26.37%) cases of type II degree injury, 19 (17.27%) cases of type III degree injury, and 5 (4.55%) cases of type IV degree injury.

**Table 1:** Baseline characteristics of the patients.

	**Data**
Age at surgery, years	68.28 ± 8.73 (45–89)
Symptom duration, months	7.03 ± 4.35 (3–20)
Sex, *n*	31 M and 79 F
Dominant arm involved, *n* (%)	85 (77.27)
Patients with trauma history, *n* (%)	82 (74.55)
Follow-up period, months	20.18 ± 5.71 (12–30)
	**No. of patients (%)**
**Lafosse classification of subscapularis tear**	
II	78 (70.91)
III	32 (29.09)
Isolated subscapularis tear	9 (8.18)
Combined rotator cuff tear	101 (91.82)
Supraspinatus	74 (73.27)
Supraspinatus and infraspinatus	27 (26.73)
**Long head of the biceps tendon**	
Normal	63 (57.27)
Complete tear	5 (4.55)
Partial tear	36 (32.73)
Dislocation or subluxation	6 (5.45)
**Typing of shoulder crest**	
I	19(17.27)
II	64(58.18)
III	27(24.55)
**Outerbridge classification of cartilage injury under arthroscopy**	
Normal	36 (32.72)
I	21 (19.09)
II	29 (26.37)
III	19 (17.27)
IV	5 (4.55)

There was a remarkable improvement in postoperative active ROM (*P* < 0.05). At the last follow-up, the forward flexion of the shoulder joint increased from 88.97 ± 26.33° to 138.38 ± 26.48° (*P* < 0 0.05), the abduction increased from 88.86 ± 25.27° to 137.78 ± 25.64° (*P* < 0 0.05), the external rotation increased from 46.37 ± 14.48° to 66.49 ± 14.15° (*P* < 0 0.05), and the internal rotation increased from 40.03 ± 9.01°to 57.55 ± 7.43° (*P* < 0 0.05). In addition, compared with the preoperative score, the functional result increased remarkably in the mean VAS, Constant score, and ASES score. The VAS score decreased from 5.05 ± 2.11 to 1.01± 0.85 (P < 0 0.05), the Constant shoulder joint score increased from 40.14 ± 15.07 to 81.75 ± 11.00 (*P* < 0 0.05), and the ASES score increased from 37.88 ± 13.24 to 82.01 ± 9.65 (*P* < 0 0.05). There were 31 male and 79 female patients with no significant difference among them (Table 2).

**Table 2:** Surgery results of range of motion and clinical results.

**Indicator**	**Total (*N* = 110)**	**Male (*N* = 31)**	**Female (*N* = 79)**	***p-*value(M vs. F)**
**Range of motion**				
**Forward flexion**				
Preoperation	88.97 ± 26.33	88.58 ± 30.82	89.13 ± 24.34	0.923
3-month postoperation	140.74 ± 22.47	137.39 ± 24.40	142.05 ± 21.53	0.332
Last follow-up	138.38 ± 26.48	140.13 ± 26.35	137.70 ± 26.50	0.668
**Abduction**				
Preoperation	88.86 ± 25.27	88.45 ± 29.28	89.03 ± 23.51	0.916
3-month postoperation	136.09 ± 24.35	139.10 ± 25.04	134.91 ± 23.97	0.422
Last follow-up	137.78 ± 25.64	138.71 ± 25.84	137.42 ± 25.55	0.814
**External rotation**				
Preoperation	46.37 ± 14.48	43.26 ± 14.24	47.59 ± 14.38	0.160
3-month postoperation	61.85 ± 12.38	63.52 ± 13.44	61.20 ± 11.88	0.383
Last follow-up	66.49 ± 14.15	68.84 ± 14.08	65.57 ± 14.07	0.280
**Internal rotation**				
Preoperation	40.03 ± 9.01	40.65 ± 8.49	39.78 ± 9.20	0.656
3-month postoperation	52.86 ± 7.7957.55 ± 7.43	52.23 ± 8.24	53.11 ± 7.59	0.594
Last follow-up		57.16 ± 6.67	57.71 ± 7.70	0.731
**VAS score**				
Preoperation	5.05 ± 2.11	5.10 ± 2.18	5.04 ± 2.08	0.896
3-month postoperation	1.98 ± 1.49	2.10 ± 1.49	1.94 ± 1.49	0.616
Last follow-up	1.01 ± 0.85	0.87 ± 0.75	1.05 ± 0.88	0.325
**ASES score**				
Preoperation	37.88 ± 13.24	39.71 ± 14.09	40.30 ± 15.44	0.854
3-month postoperation	75.29 ± 14.43	74.65 ± 14.30	72.66 ± 14.47	0.521
Last follow-up	82.01 ± 9.65	81.35 ± 12.16	81.91 ± 10.51	0.813
**Constant score**				
Preoperation	40.14 ± 15.07	38.58 ± 12.48	37.61 ± 13.52	0.732
3-month postoperation	73.22 ± 14.45	76.26 ± 14.36	74.91 ± 14.44	0.663
Last follow-up	81.75 ± 11.00	81.97 ± 11.01	82.03 ± 9.07	0.978

Data are expressed as the mean ± SD (standard deviation). VAS, visual analog scale; ASES, American Shoulder and Elbow Surgeons. Compared with preoperation, p < 0.05. Compared with 3-month postoperation, p < 0.05.

**Figure 3:**
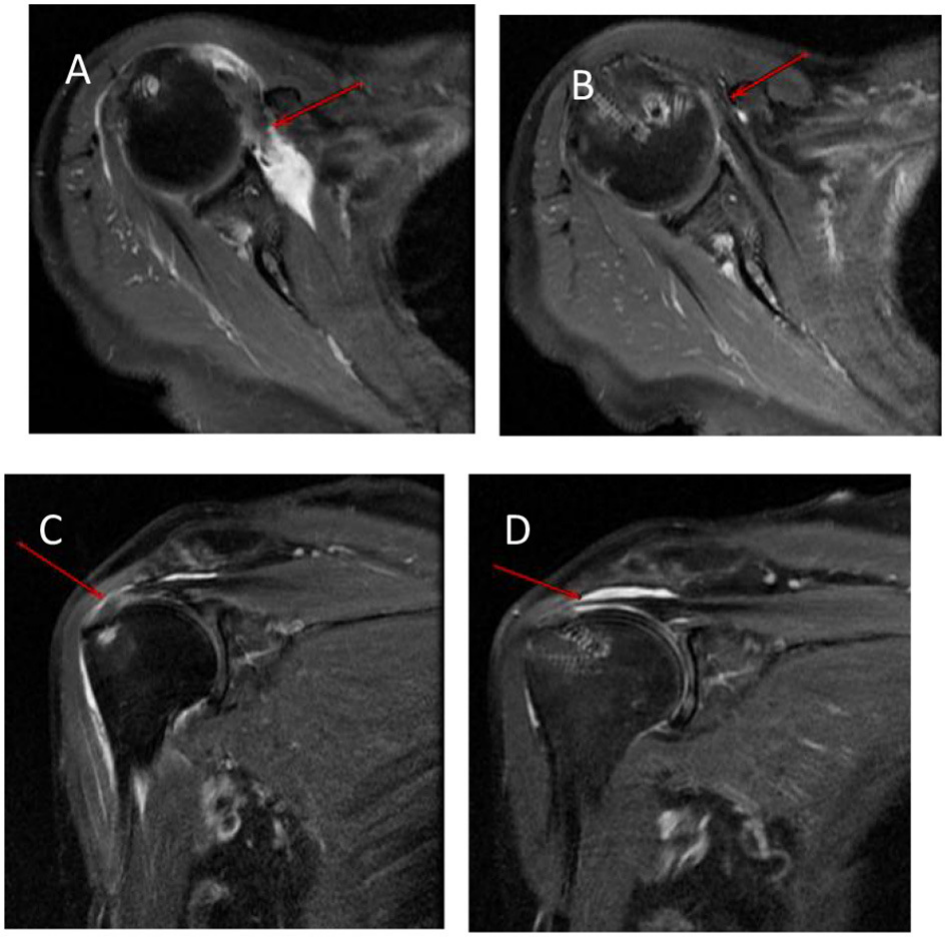
Preoperative and postoperative MRI scans of the right shoulder in a 71-year-old female patient. **(A)** An axial T2-weighted MRI scan shows a chronic subscapularis tear (arrows) preoperatively. **(B)** Complete structural integrity of the repaired subscapularis tendon (arrows) is shown on axial T2-weighted MRI at 12 months postoperatively. **(C)** A coronal T2-weighted MRI scan shows a combined supraspinatus tear. **(D)** Complete structural integrity of the repaired supraspinatus tendon is shown on coronal T2-weighted MRI at 12 months postoperatively.

Some patients' complete structural integrity of the repaired subscapularis tendon was observed *via* MRI after surgery (Figure 3). Among the 2 (1.81%) patients with subscapularis re-tears, one had partial-thickness re-tears and the other had full-thickness re-tears. Preoperatively, the two patients had combined full-thickness supraspinatus and infraspinatus tears. After repair, the 3 tendons were all re-torn. Both of these patients with re-tear had Goutallier grade III fatty infiltration. The patients with Goutallier grade I or II fatty infiltration had no re-tear. Therefore, we speculated that the rate of re-tear in patients with grade III fat infiltration would be relatively high, but further research was still needed. No postoperative complications such as neurovascular injury or infection were found in the follow-up.

## Discussion

The subscapularis is the main driving force for the internal rotation of the shoulder joint and an important structure for maintaining horizontal couple balance and dynamic stability of the shoulder joint (19). With the application of arthroscopy and the improvement of imaging technology, the diagnosis and treatment rate of subscapular tendon tears have significantly improved, and the patient's pain has been reduced (20, 21). However, isolated subscapular tendon injuries or combined rotator cuff injuries are relatively rare, and their treatment methods are controversial (22, 23). Arthroscopic minimally invasive technology can effectively repair tears in the subscapular muscle. However, good suture techniques are the foundation for the healing of the subscapular tendon and the recovery of shoulder joint function. The techniques for repairing subscapular muscle tears under arthroscopy mainly include single-row sutures, double-row suture, and suture bridges. Different suture techniques have their own advantages and disadvantages. The traditional single-row anchor nail technique, as the most basic method for arthroscopic repair of shoulder sleeves, involves point contact tendon–bone healing, which is difficult to achieve anatomically and is prone to stress concentration and suture cutting, leading to repair failure. Moreover, due to the narrow anterior space of the shoulder joint, it is relatively difficult to operate using traditional single-row anchor nail techniques (24). The double-row anchor nail technology can reduce the stress on the tendon–bone healing surface and have greater biomechanical strength. However, the difficulty of operating the double-row anchor technique has increased as multiple anchors exist on the tendon–bone healing surface, resulting in a reduction in the healing area. The increase of thread knots on the surface of the shoulder sleeve causes postoperative adhesion or the formation of new impacts (25). At present, suture bridge technology is the most commonly used application, which uses several tail lines to cross and form a grid-like structure to compress the tendon, reduce the tendon–bone gap, and achieve a better fixation effect than other fixation methods. At the same time, it avoids joint fluid infiltration into the tendon–bone gap, further providing a good healing environment for tendon–bone healing (26). However, the suture bridge technology also faces disadvantages such as difficulty in operation, long surgical time, and the high cost of multiple anchor nail surgeries. Yoon et al. (27) compared the clinical efficacy and structural integrity of the single-row suture technique and the double-row suture bridge technique under arthroscopy for repairing a full-thickness tear of the subscapular tendon. At the 2-year follow-up after surgery, both groups showed significant improvement in clinical outcomes. However, none of these clinical outcome indicators showed significant differences between groups. Following up on Magnetic Resonance Angiography (MRA) or Computed Tomography Angiography (CTA), there was no significant difference in the overall re-tearing rate between the single-row group (13%, 4 out of 31) and the double-row group (12%, 3 out of 25) (27). This indicates that the single-row technology can also achieve dual-row treatment effects.

The single-row repair technology essentially involves two FiberWire^®^ sutures weaving techniques, where the suture forms two knotted locking loops at the broken end of the subscapular tendon and a SwiveLock^®^ C anchor nail that fixes the broken end of the subscapular tendon to the cortical bone area outside the healing area of the small nodules. This suture technique overcomes many problems associated with the above-mentioned repair techniques. During suturing, a traction line can be pre-inserted to facilitate stretching and adjusting the tension of the retracted subscapular tendon. While ensuring appropriate tension, threading two FiberWire^®^ sutures through the broken end of the subscapular tendon and tying the knot into two locking rings forms a “double loop lasso” that disperses the cutting force of the suture on the tendon, avoiding stress concentration at a single suture point that can cause the suture to cut the tendon. Mitchell et al. (28) studied the posterior horn of the medial meniscus of the cadaver knee joint and used a similar “double locking ring” suture technique to suture the medial meniscus. Biomechanics indicates that “the double locking ring” can immediately obtain the same failure load as the natural posterior angle of the medial meniscus after suturing (28). Additionally, under the fixation of two FiberWire^®^ sutures with one SwiveLock^®^ C anchor, the scapular tendon tissue between the two locking rings forms a “face-to-face” contact with the “footprint area,” completing complete coverage of the footprint area and uniform compression of the tendon–bone healing area. Seppel et al. (29) used the Mason–Allen technique to repair the subscapular tendon, using an average of two (1–3) anchor screws (29). For patients with less severe fat infiltration and tendon retraction, we used one anchor nail, which is a simple and cost-effective procedure. Jeong et al. (30) believed that, after the tear of the subscapular tendon, it retracts inward and downward, so the optimal point for anchor screw implantation is the outer upper corner of the footprint area (30). Dyrna et al. (31) found that adding one anchor nail to the outer upper corner of the footprint area of the subscapularis muscle significantly improves biomechanics after repair in recent biomechanical studies (31). We also used the SwiveLock^®^ anchor to fix in this position, which not only maximizes the coverage of the footprint area but also maintains the contact pressure between the tendon and bone, promoting tendon–bone healing and achieving good clinical results.

Arthroscopic repair of a subscapular muscle tear resulted in significant improvement in the ROM and functional score of the shoulder joint. Traditional open surgery has some drawbacks, such as large incisions, postoperative pain, and difficulty in postoperative recovery. However, arthroscopic minimally invasive surgery not only overcomes the shortcomings of open surgery but can also simultaneously treat other rotator cuff tendons. Nové-Josserand et al. (32) compared and analyzed 13 cases of traditional open surgery group and 22 cases of minimally invasive surgery under endoscopy. The arthroscopic group achieved a tendon healing rate of 86% after surgery, and the Constant score increased from 66 points before surgery to 85 points after surgery (32). According to the meta-analysis conducted by Mall et al. (33), there was no significant difference in the Constant score, mobility, or postoperative healing rate between traditional open surgery and arthroscopic minimally invasive surgery. However, arthroscopic minimally invasive surgery has the characteristics of small incision, reduced postoperative pain, and easy postoperative recovery (33). Jeong et al. (30) evaluated the clinical efficacy of the arthroscopic single-row mattress suture technique for repairing isolated or combined rotator cuff tears. The average follow-up was 29.5 ± 4.0 months. The study showed that the Constant score (preoperative 50.3 ± 21.0 vs. postoperative 75.7 ± 16.6) and ASES score (preoperative 46.6 ± 18.3 vs. postoperative 81.3 ± 18.1) were significantly improved, and the differences between postoperative and preoperative were statistically significant (*p* < 0.05) (30). In our research, the single-row anchor technology uses SwiveLock^®^ C anchor and FiberWire^®^ sutures to repair isolated subscapular tendon injury or those combined with other rotator cuff tears. The clinical effect is satisfactory, which is consistent with the above research results. Repairing the injury of the subscapular muscle alone or in combination with repairing the injury of the supraspinatus and infraspinatus muscles not only improves the active internal rotation range of the shoulder joint but also shows satisfactory results for the lifting and external rotation function of the shoulder joint. Our experience is as follows: During the surgery, it is necessary to fully release the adhesion site of the subscapularis muscle and evaluate the tension of the subscapularis muscle. When suturing and pointing, we suggest attempting to enter perpendicular to the surface of the subscapularis muscle as much as possible and accurately locate the suture site to avoid repeated punctures that may cause iatrogenic tendon damage. It is necessary to ensure that the anchor insertion point is located at the outer edge of the “footprint” while ensuring appropriate tension of the subscapular tendon.

Most rotator cuff repair surgeries can fully restore the normal footprint insertion point of the tendon anatomically, but only complete tendon–bone healing can provide effective mechanical strength. Tendon–bone healing can be divided into direct healing and indirect healing, and it takes 1 year or even longer to form a typical four-layer structure. After rotator cuff repair, the tendon–bone interface cannot be completely reconstructed in the early stage but is more for scar healing, and the strength will be reduced compared to the normal stop point (34). In our study, two patients were confirmed with re-tearing of the subscapularis muscle, including an elderly patient and a patient engaged in heavy work. At the patient's last follow-up, the ROM and muscle strength of the shoulder joint were still poor, and the pain in the shoulder joint was significantly relieved compared with preoperation. Both patients had severe tearing of the supraspinatus and infraspinatus muscles. On the contrary, there was no further tearing for patients with isolated subscapular tears or only combined supraspinatus tears. For massive rotator cuff injury, some scholars believe that it can be completely repaired through thorough release under the microscope and foot imprint internal transfer technology. If there is severe retraction and fat infiltration of rotator cuff tissue, a partial repair can be used to restore shoulder joint function. However, other scholars believe that, when there is Lafosse V-type subscapular muscle injury, suturing should be abandoned (35). Yoon et al. (36) showed that there was no significant difference in clinical outcomes between non-surgical and surgical treatment for isolated subscapular tendon tears with advanced fat infiltration. Surgical treatment is not recommended because there is still a high rate of re-tearing after surgical repair. It is recommended to detect and repair the tear of the subscapular tendon promptly before the lesion progresses to Goutellier grade III or IV fat infiltration, to achieve better clinical results (36). In two patients with a full layer tear of the subscapular muscle combined with injury to the supraspinatus and infraspinatus muscles in this group, satisfactory results were not achieved after repair using the footprint transfer technique. We believe that, for patients with Lafosse V subscapular muscle injury combined with upper rotator cuff injury, severe retraction, and Goutellier ≥3, suturing should be abandoned and a more suitable alternative treatment method should be chosen, such as total shoulder arthroplasty.

Similar to other rotator cuff repairs, the purpose of repairing subscapular tendon tears is to alleviate pain, achieve good tendon–bone healing, and prevent further tearing. There is currently a lot of debate about the role of coracoid process formation in the re-tearing of the subscapular tendon. Çetinkaya et al. (38) indicate that, based on MRI, coracoid process reconstruction should be performed if the distance between the coracoid and humerus is < 6–7 mm (37). However, due to significant visual errors, it is difficult for us to accurately identify the distance between the coracoid and humerus as 6–7 mm in clinical work. The study by Ulunay et al. (38) showed that there were no significant differences in coracoid process parameters such as coracohumeral distance (CHD) and coracoid overlap (CO) between isolated subscapular tendon tears and the healthy side (38). Therefore, our case did not undergo routine coracoid process reconstruction surgery. In addition, there is also controversy regarding the treatment of the long-head tendon of the biceps brachii muscle. Research has shown the long head tendon of the biceps brachii muscle loses stability and is prone to cutting the subscapular tendon, leading to tearing after injury (39). In our case, there were five patients with spontaneous rupture of the long head tendon of the biceps brachii muscle without special treatment. Overall, 42 patients (38.18%) had injury or dislocation of the long head tendon of the biceps brachii muscle, of which 31 older patients underwent biceps tendon amputation and 11 younger or exercise-demanding patients underwent tendon fixation with suture anchors. Our study suggests that the presence of injury to the long head tendon of the biceps brachii muscle in patients is an independent influencing factor for postoperative efficacy. However, if attention is paid to the management of combined injuries of the long head tendon of the biceps brachii muscle during surgery, postoperative shoulder joint function can be significantly improved for either simple cutting or cutting fixation, which is basically consistent with the research results reported by Aflatooni et al. (40). According to research, the rate of re-tearing after repair of the subscapular tendon is ~10% (41). In this study, isolated subscapular muscle tears were less common (8.18%), and most cases were combined supraspinatus and/or infraspinatus muscle tears (91.82%). The rate of subscapular tendon re-tears is 7.1%, which is relatively lower than many similar reports. The reason for this is that the postoperative MRI examination may be false negative and further research is needed to confirm whether the long head of the biceps brachii tendon resection surgery is beneficial for reducing the rate of re-tearing.

### Limitations

SwiveLock^®^ C anchor and FiberWire^®^ suture repair of subscapular tendon tears under arthroscopy can achieve good clinical results and tendon–bone healing. However, there are also some shortcomings in this study: ① the cases were not grouped and observed based on the size of the subscapular muscle tear, and Lafosse type IV and V cases have not been studied yet. It has not yet been possible to evaluate the suture effect of this suture method on giant subscapular tendon tears. ② The incidence of isolated subscapular tears is relatively low, and postoperative functional recovery is not only affected by the subscapular tendon but also by the healing of the supraspinatus and/or infraspinatus tendons. This study did not fully consider the impact of other rotator cuff repairs on shoulder joint function. ③ This is a retrospective study, which has inherent drawbacks, including the possibility of bias in data collection and organization. However, the data were collected from patients treated by surgeons at the same hospital. ④ No control trial group was set up, and the follow-up time was relatively short.

## Conclusion

In this study, we repaired the subscapularis muscle with a single-row technique fixed by SwiveLock^®^ C anchor and FiberWire^®^ sutures and evaluated its efficacy. The results indicate that the single-row repair procedure is simple in operation, significantly improves clinical efficacy, and has good tendon integrity. This is an effective method for arthroscopic repair of the subscapular tendon.

## Data availability statement

The raw data supporting the conclusions of this article will be made available by the authors, without undue reservation.

## Ethics statement

The studies involving human participants were reviewed and approved by the Ethics Committee of The Third Affiliated Hospital of Guangzhou University of Chinese Medicine [No. KY (2019) 002]. The patients/participants provided their written informed consent to participate in this study.

## Author contributions

WZ, RW, and HW contributed to the conception and design. HW contributed to administrative support. WZ and XW contributed to the manuscript writing. RW contributed to the revised manuscript. ZLiu, ZLiao, SC, ZY, and XH contributed to the collection and assembly of data. XW and SZ contributed to the data analysis. All authors read and approved the final manuscript.
